# Hernandezine, a novel AMPK activator induces autophagic cell death in drug-resistant cancers

**DOI:** 10.18632/oncotarget.6980

**Published:** 2016-01-22

**Authors:** Betty Yuen Kwan Law, Simon Wing Fai Mok, Wai Kit Chan, Su Wei Xu, An Guo Wu, Xiao Jun Yao, Jing Rong Wang, Liang Liu, Vincent Kam Wai Wong

**Affiliations:** ^1^ State Key Laboratory of Quality Research in Chinese Medicine, Macau University of Science and Technology, Macau, China

**Keywords:** hernandezine, AMPK activator, autophagy, autophagic cell death, drug-resistant cancer

## Abstract

Drug resistance hinder most cancer chemotherapies and leads to disease recurrence and poor survival of patients. Resistance of cancer cells towards apoptosis is the major cause of these symptomatic behaviours. Here, we showed that isoquinoline alkaloids, including liensinine, isoliensinine, dauricine, cepharanthine and hernandezine, putatively induce cytotoxicity against a repertoire of cancer cell lines (HeLa, A549, MCF-7, PC3, HepG2, Hep3B and H1299). Proven by the use of apoptosis-resistant cellular models and autophagic assays, such isoquinoline alkaloid-induced cytotoxic effect involves energy- and autophagy-related gene 7 (Atg7)-dependent autophagy that resulted from direct activation of AMP activated protein kinase (AMPK). Hernandezine possess the highest efficacy in provoking such cell death when compared with other examined compounds. We confirmed that isoquinoline alkaloid is structurally varied from the existing direct AMPK activators. In conclusion, isoquinoline alkaloid is a new class of compound that induce autophagic cell death in drug-resistant fibroblasts or cancers by exhibiting its direct activation on AMPK.

## INTRODUCTION

Autophagy is a highly coordinated process responsible for maintaining normal cellular homeostasis under nutrient deprivation conditions. This process involves the lysosomal degradation of cellular components such as misfolded proteins or damaged organelles. Defects in autophagy are correlated to the pathogenesis of diseases such as cancer, myopathy and neurodegeneration [1]. AMP activated protein kinase (AMPK), maintains normal energy balance by regulating cellular metabolisms in an AMP/ADP ratio-dependent manner, is responsible for the proper mechanistic modulation of autophagy [2]. During cellular starvation, AMPK induces autophagy by phosphorylating Ulk1, the mammalian counterpart of ATG1, at Ser 317 and 777 [3, 4]. Molecular studies demonstrated that Ulk1 together with another mammalian ATG1 homolog, Ulk2, form complex with mATG13 and FIP200 (mammalian homologues of ATG13 and ATG17) and regulate the autophagic machinery [5, 6]. Yeast models have also suggested the inductive role of ATG1 kinase in autophagy [7]. Under nutrient-rich conditions, the activation of mTOR prevents the phosphorylation of Ulk1 activation through Ser 757, which finally inhibits the Ulk1-AMPK dependent induction of autophagy [4]. AMPK stimulates autophagy through the inhibition of mTORC1, which is the key regulator of growth factor and nutrient signals transduction [4]. Recently, small-molecule AMPK activators have been identified as potential therapeutic agent for metabolic diseases or cancers [2, 8, 9]. Natural compounds such as α-Lipoic acid, polyphenols (resveratrol) and isoquinoline alkaloid (berberine); small molecule activators such as A-769662, metformin, thiazolidinediones (TZDs) and alkyl benzoquinones could directly or indirectly activate AMPK in a variety of cell types [10, 11]. In fact, autophagy may play its anti-cancer role by preventing accumulation of damaged proteins and organelles which lead to the progression of tumor growth [12], or via the induction of autophagic cell death [13].

Hernandezine, an alkaloid isolated from Chinese medicinal herb, has long been used for treating hypertension and angina pectoris [14, 15]. There was report suggesting hernandezine blocks the influx of calcium via non selective cation channels in HL-60 cells [16]. Further study showed that the calcium influx triggered by depletion of internal calcium stores was blocked by hernandezine [17]. In the present study, we depicted the role of hernandezine in inducing autophagy and autophagic cell death in apoptosis-resistant cells via the direct activation of AMPK.

## RESULTS

### Hernandezine exhibits specific cytotoxicity towards cancer cells

**Figure 1:**
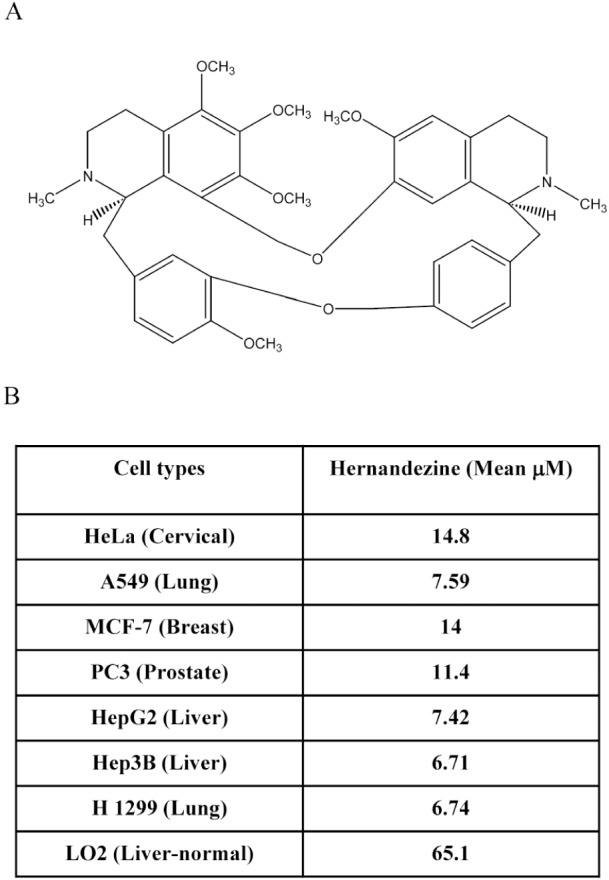
Cytotoxicity of hernandezine (**A**) Chemical structure of hernandezine. (**B**) Hernandezine exhibited specific cell cytotoxicity towards a panel of cancer and normal cells. The IC_50_ values shown on the chart were the means of three independent experiments.

We previously demonstrated that a group of alkaloid compounds including liensinine, isoliensinine, dauricine and cepharanthine exhibit potent anti-cancer effect via autophagy induction [13]. Hernandezine, an alkaloid isolated from *Thalictrum glandulosissimum* sharing structural similarity with isoquinoline alkaloids (Figure 1A), may also possess potent anti-cancer efficacy. To investigate the anti-cancer effect of hernandezine, a panel of cancer cells, including HeLa (cervical), A549 (lung), MCF-7 (breast), PC3 (prostate), HepG2 (liver), Hep3B (liver) and H1299 (lung) were adopted in the cytotoxicity assay, whereas normal human hepatocytes, LO2, were used for comparison. As shown in Figure 1B, hernandezine demonstrated potent cytotoxic effects towards all these cancer cells types, especially on A549 lung cancer (mean IC_50_, 7.59 μM), HepG2 liver cancer (mean IC_50_, 7.42 μM), Hep3B liver cancer (mean IC_50_, 6.71 μM) and H1299 lung cancer (mean IC_50_, 6.74 μM). In contrast, hernandezine exhibited relative low cytotoxicity towards normal liver hepatocytes, LO2 (mean IC_50_, 65.1 μM), suggesting that its specific cytotoxic effect towards cancer cells.

### Hernandezine induces autophagic GFP-LC3 puncta in various types of cancer cells

**Figure 2:**
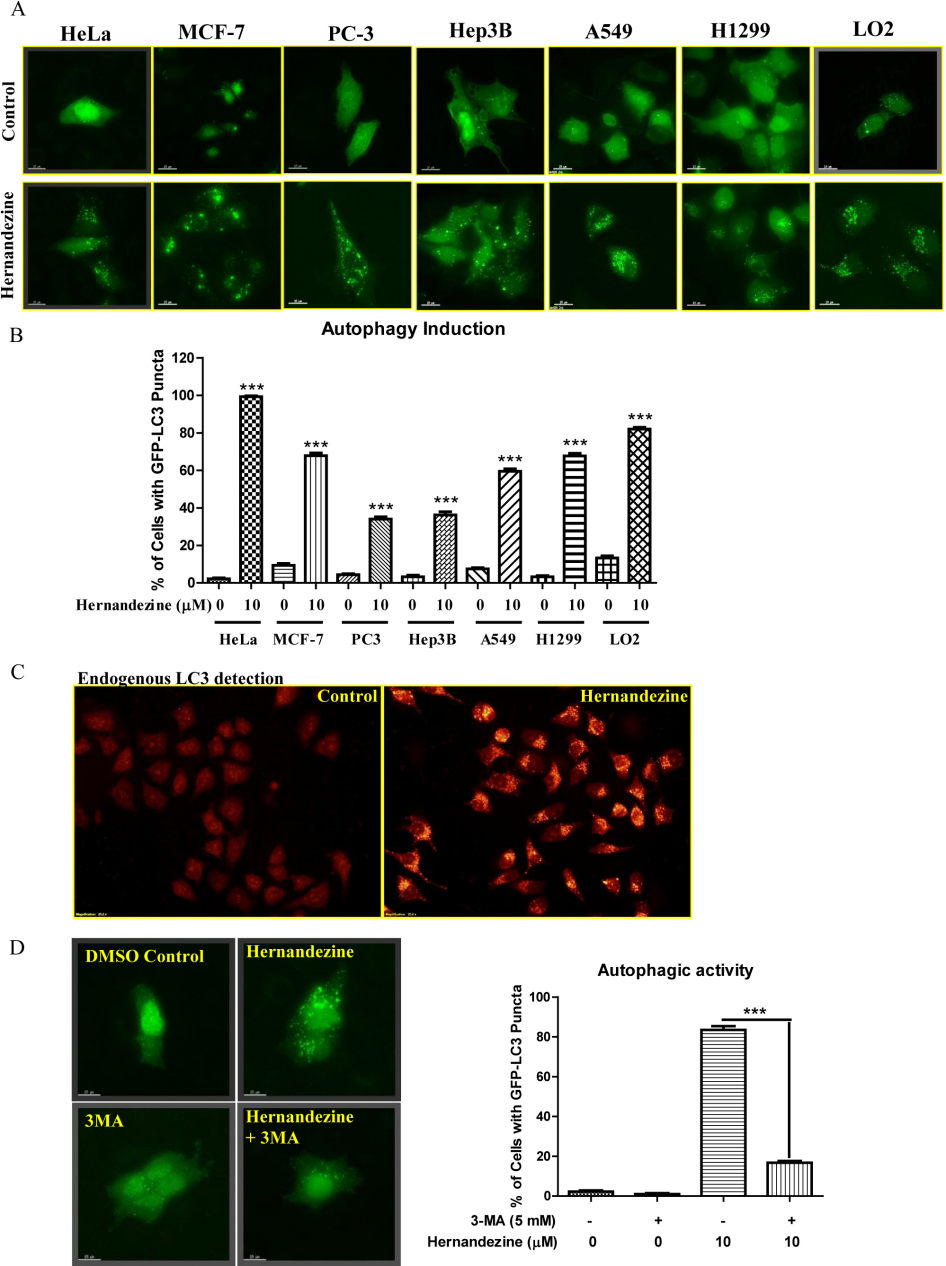
Hernandezine induced autophagy in a panel of cancer and normal cells (**A**) Detection of hernandezine-induced GFP-LC3 puncta formation in HeLa, MCF-7, PC3, Hep3B, A549, H1299 cancer cells and LO2 normal hepatocytes. Cells were transiently transfected with the EGFP-LC3 plasmid for 24 h and then treated with DMSO (−ve Ctrl) or 10 μM of hernandezine for an additional 24 h. Fluorescence images were captured at 60 × magnification; scale bar, 15 mm. (**B**) Bar chart represented the quantitation of autophagic cells. (**C**) Endogenous expression of LC3-II in HeLa cells. HeLa cells treated with 10 μM of hernandezine for 24 h were visualised by fluorescence microscopy after staining with the LC3-II and TRITC-conjugated anti-mouse secondary antibody. (**D**) Autophagic inhibitor 3-MA abrogated hernandezine-mediated autophagy. HeLa cells were transiently transfected with the GFP-LC3 plasmid for 24 h and then treated with DMSO (Ctrl) or hernandezine (10 μM) with or without 5 mM of 3-MA for 24 h. Representative micrographs of cells with GFP-LC3 puncta formation and bar charts with the quantitation of autophagic cells were shown. Data represented the means of three independent experiments. Error bars, S.D. ***P < 0.001 for hernandezine-treated cells with and without 3-MA. Fluorescence images were captured at 60 × magnification; scale bar, 15 μm.

To confirm whether hernandezine is capable of inducing autophagy in variety of cancer cells, we utilized HeLa, MCF-7, PC-3, Hep3B, A549 and H1299, and LO2 normal human hepatocytes for detecting the autophagic GFP-LC3 puncta. As shown in Figure 2A, 10 μM of hernandezine induced GFP-LC3 puncta formation in all the cancer cells and normal hepatocytes, indicating the autophagic effect of hernandezine is not cell-type specific. However, quantitation of the percentages of cells with autophagic puncta formation showed that, different cancer cell types possess different potency for autophagy induction in response to hernandezine treatment (Figure 2B). In addition, the formation of LC3-II puncta was further verified by immunofluorescence staining against endogenous LC3-II in HeLa cancer cells (Figure 2C). Besides, the hernandezine-induced autophagic effect was further validated with 3-methyladenine (3-MA), a well-known PI3K inhibitor commonly used to inhibit autophagy [18]. As demonstrated by the decreased percentage of cells with GFP-LC3 puncta formation (Figure 2D), addition of 3-MA abrogated hernandezine-induced autophagy.

### Hernandezine induces autophagic flux in HeLa cancer cells

**Figure 3:**
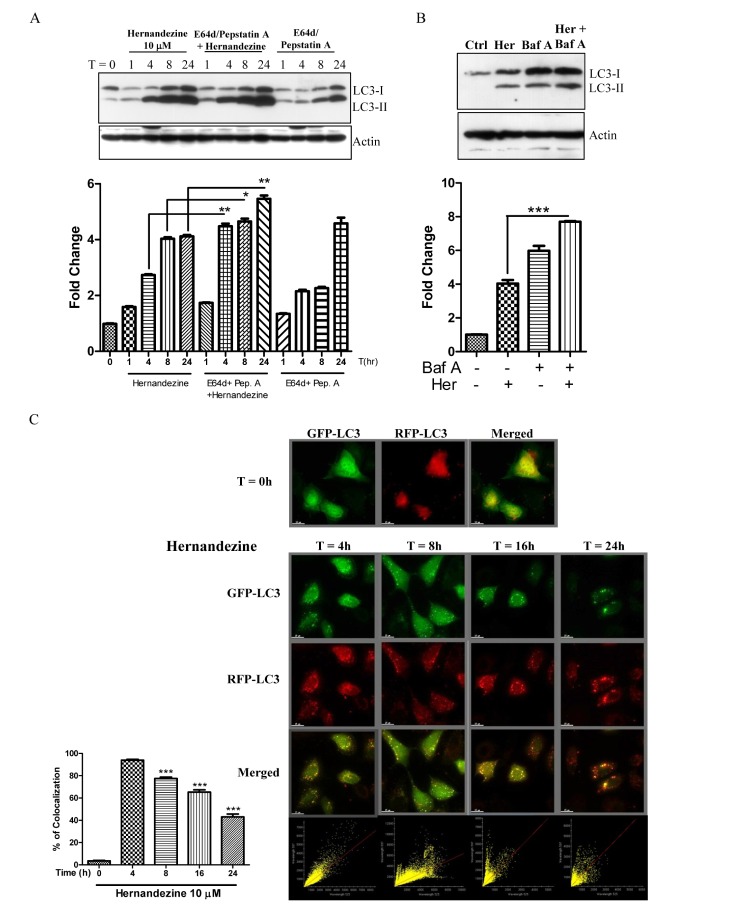
Hernandezine induced autophagic flux in HeLa cancer cells (**A** and **B**) Hernandezine induced LC3-II conversion in the presence of lysosomal inhibitors. HeLa cells were treated with 10 μM of hernandezine in the presence or absence of 10 mg/mL lysosomal protease inhibitors (E64d and pep. A) for 24 h, or 50 nM bafilomycin A for 8 h. Cell lysates were analysed by western blot for LC3 conversion (LC3-I, 18 kDa; LC3-II, 16 kDa) and β-actin. LC3-II band intensities were quantified using densitometric analysis and normalised to β-actin. Data were expressed as a fold change relative to the DMSO-treated negative control. Bar charts were representatives of three independent experiments. (**C**) mRFP-GFP-LC3 fluorescence localisation pattern of hernandezine. HeLa cells were firstly transfected with the mRFP-GFP-LC3 plasmids for 24 h and then treated with 10 μM of hernandezine for 0–24 h. Cells were then subjected to immunocytochemical analysis and the mRFP^+^–GFP^+^ (yellow) puncta were scored; scale bar, 15 mm. Each correlation plot was derived from the field shown in the immunofluorescence image. The colocalisation of mRFP with GFP signal from tfLC3 puncta was measured using ImageJ software. The percentage of colocalisation was displayed in the bar chart. The values indicated the average of at least five images. Error bars, S.D., ***P < 0.001.

Induction of autophagy indicated by an increased formation of GFP-LC3 puncta using fluorescence microscopy, or LC3 lipidation using western blot, can be resulted from either an induction of autophagic flux or failure in fusion of autophagosomes and lysosomes. Hence, we measured the conversion of soluble LC3-I to lipid-bound LC3-II in the presence of E64d and pepstatin A, which inhibit lysosomal proteases including cathepsins B, D and L; or bafilomycin, which inhibits the fusion of autophagosome and lysosome by raising lysosomal pH [19, 20]. As expected, hernandezine increased the rate of LC3-II formation in the presence of the inhibitors when compared with the use of inhibitors or hernandezine alone (Figure 3A and 3B). This result suggested that hernandezine induced autophagic activity through enhanced autophagic flux and autophagosome formation.

We further monitored the autophagic flux using mRFP-GFP tandem fluorescent-tagged LC3 (tfLC3) plasmid. Given that the localisation pattern of GFP-LC3 and tfLC3 are different, the LC3 fusion construct with both red (mRFP) and green (GFP) fluorescence proteins is therefore widely used for detection of autophagosomes [21]. Due to the difference in the stability of GFP and mRFP under different pH conditions [22], acidic environment of lysosome will quench the GFP signal but not the mRFP signal. Therefore, we overexpressed the tfLC3 plasmid to monitor autophagic flux. As shown in Figure 3C, while the yellow merged image (mRFP+-GFP+) represents the autophagosomes, merged images with red puncta (mRFP+-GFP−) indicates autophagic flux with the formation of autolysosomes [21]. Our results demonstrated a time-dependent decrease in the percentage of cells with mRFP-GFP colocalisation after hernandezine treatment, confirming the induction of autophagic flux by this alkaloid in HeLa cancer cells.

### Hernandezine activates AMPK kinase for induction of autophagy and cell death

**Figure 4:**
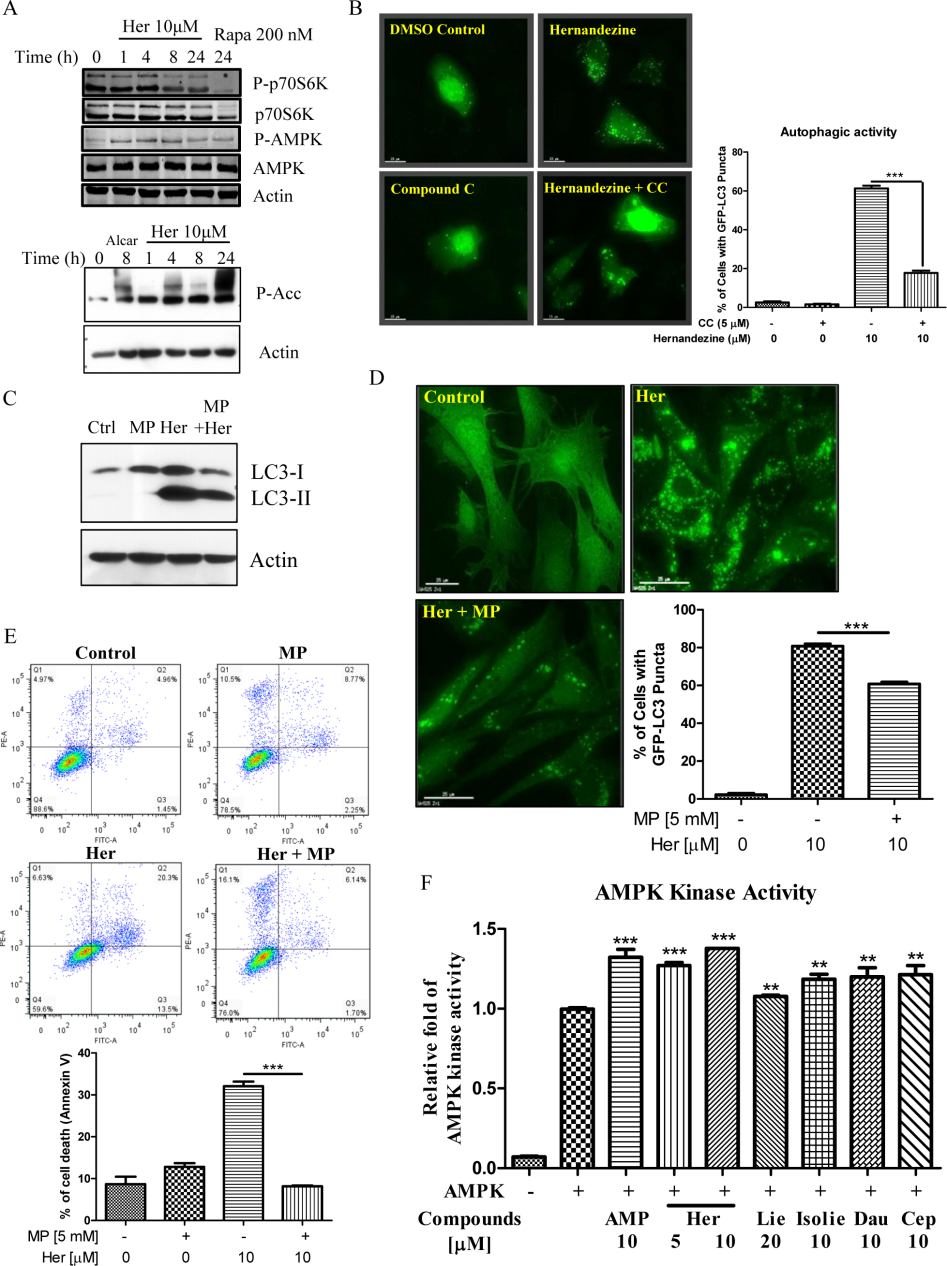
Role of the AMPK-mTOR signalling cascade in hernandezine-induced autophagy (**A**) Hernandezine activated AMPK-mTOR signalling pathways. HeLa cells treated with 10 μM of hernandezine for 0–24 h were analysed for p-AMPK, total AMPK, p-p70S6K, total p70S6K, P-Acc and β-actin. Rapamycin (Rapa, 200 nM) or Alcar (1 mM) were used as the positive control. (**B**) AMPK inhibitor abrogated the hernandezine-mediated autophagic effect in cancer cells. HeLa cells were transiently transfected with the EGFP-LC3 plasmid for 24 h, and then treated with DMSO (Ctrl) or 10 μM of hernandezine with or without 5 μM of the AMPK inhibitor compound C (CC) for 24 h. The cells were counted at 60X magnification; scale bar, 15 mm. Bar chart represented the quantitation of autophagic cells with GFP-LC3 puncta. Data represented the means of three independent experiments. Error bars, S.D. ***P < 0.001 for hernandezine-treated cells with and without CC. (**C**) Methyl pyruvate blocked the hernandezine-induced LC3-II conversion. HeLa cells were treated with 10 μM of hernandezine (Her) in the presence or absence of 5 mM of methyl pyruvate (MP) for 24 h. (**D**) Methyl pyruvate decreased the hernandezine-mediated autophagic effect in cancer cells. HeLa cells were transiently transfected with the EGFP-LC3 plasmid for 24 h and then treated with DMSO (Ctrl) or 10 μM of Her with or without 5 mM of MP for 24 h. The cells were counted at 60 × magnification; scale bar, 15 mm. Bar chart represented the quantitation of autophagic cells with GFP-LC3 puncta. Data were the means of three independent experiments; error bars, S.D. ***P < 0.001. (**E**) Methyl pyruvate abolished the hernandezine-induced cell death in cancer cells. HeLa cells were incubated with DMSO (Ctrl), or 10 μM of Her with or without 5 mM of MP for 24 h. Hernandezine-induced cell death in HeLa cells was then measured by flow analysis after annexin V staining. Data from the bar chart represented the means ± S.D. of cell death (%) from three independent experiments. (**F**) Hernandezine directly targeted and activated AMPK kinase activity. AMP was positive control, 20 μM liensinine (Lie) and 10 μM of isoliensinine (Isolie), dauricine (Dau) and cepharanthine (Cep) are alkaloid compounds used for comparison with hernandezine.

AMPK is a sensor of cellular energy status and is activated under high intracellular AMP conditions such as hypoxia or nutrient deprivation, thereby induces autophagy via the mTOR-dependent pathway [23]. Phosphorylation of AMPK and its downstream target Acetyl-Coenzyme A Carboxylase (ACC) are required for small-molecule induced autophagy [24]. As demonstrated by western blot analysis, AMPK phosphorylation was increased in response to hernandezine treatments (Figure 4A). The phosphorylation was accompanied by a reduction in phosphorylated p70S6K, a downstream target of mTOR (Figure 4A). Concomitantly, ACC, the direct downstream target of AMPK, was phosphorylated upon hernandezine treatments (Figure 4A, lower panel). In addition, a decrease in hernandezine-induced GFP-LC3 autophagic puncta formation was observed in cells pre-treated with the AMPK inhibitor compound C (CC) (Figure 4B), suggesting the involvement of AMPK signalling in hernandezine-induced autophagy. Alternatively, supplementation of glycolytic intermediate, methyl pyruvate (MP), was able to suppress hernandezine-induced LC3-II conversion and GFP-LC3 puncta formation (Figure 4C and 4D), suggesting hernandezine-induced autophagy involved energy depletion. To address whether hernandezine-induced cell death is due to energy depletion, we examined its cytotoxicity with the presence of methyl pyruvate using annexin V flow cytometry. As shown in Figure 4E, while hernandezine induced cell death in HeLa cancer cells, the addition of methyl pyruvate abolished the compound-induced cell death. Most importantly, cell-free AMPK kinase assay has revealed that liensinine, isoliensinine, dauricine, cepharanthine and hernandezine, could activate the AMPK kinase activity dose-dependently (Figure 4F). All these evidence suggested that isoquinoline alkaloid activates on AMPK kinase directly.

### Hernandezine-induced autophagy promotes cell death

**Figure 5:**
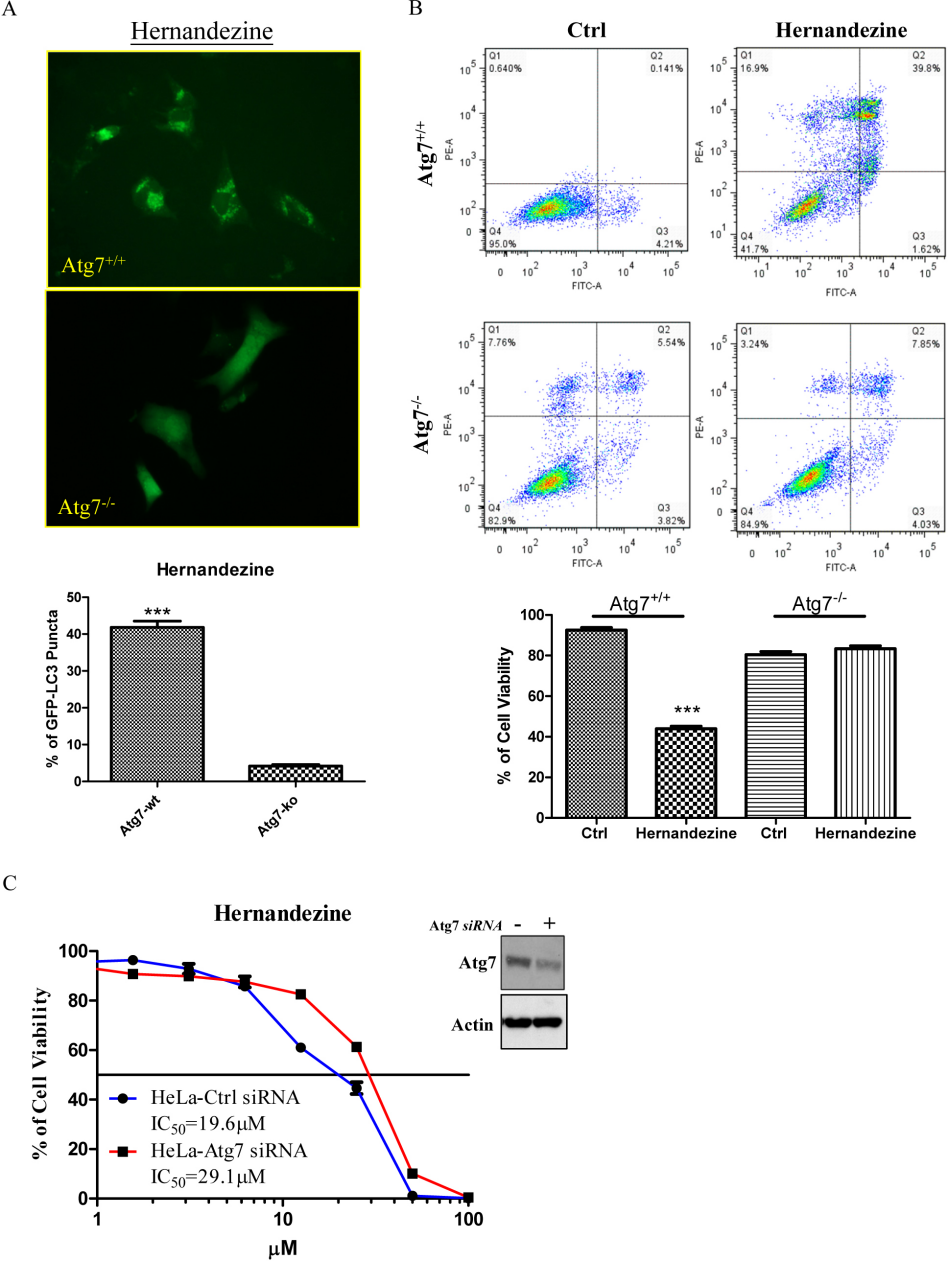
Hernandezine induced autophagy and cell death in Atg7 wild-type and deficient MEFs (**A**) Both Atg7^+/+^ wild-type and Atg7^−/−^ deficient MEFs were transiently transfected with the EGFP-LC3 plasmid for 24 h and then treated with DMSO (Ctrl) or 10 μM of hernandezine for 24 h. The cells were then fixed for fluorescence imaging and scoring. Bar chart represented the quantitation of autophagic cells. ***P < 0.001, compared between Atg7^+/+^ wild-type and Atg7^−/−^ deficient MEFs. (**B**) Annexin V flow cytometry analysis of hernandezine in Atg7+/+ wild-type and Atg7^−/−^ deficient MEFs. Both Atg7 wild-type and deficient MEFs were incubated with DMSO (Ctrl) or 10 μM of hernandezine for 24 h. Hernandezine-induced cell death was then measured by flow analysis after annexin V staining. Data from the bar chart represented the means ± S.D. of cell viability (%) from three independent experiments. (**C**) Cytotoxicity of hernandezine in HeLa cancer cells with *siRNA* knockdown of Atg7. HeLa cancer cells with or without Atg7 *siRNA* knockdown were incubated with hernandezine for 72 h, MTT assay was performed to determine their cytotoxicity. Western blot indicated the expression of Atg7 in HeLa cancer cells with *siRNA* knockdown of Atg7. The IC_50_ values shown on the chart were the means of three independent experiments.

Autophagy-related gene 7 (Atg7) is essential for vesicle nucleation and elongation during autophagy [20]. Previous studies showed that Atg7-knockout mice die due to their failure in adaptation of neonatal starvation [25]. Meanwhile, cancer cells lacking Atg7 gene are insensitive to small-molecules-induced autophagy [13, 26]. To determine whether hernandezine requires Atg7 for autophagy induction, GFP-LC3 transfected Atg7-wild-type and -deficient mouse embryonic fibroblasts (MEFs) were incubated with hernandezine for 24 h. The hernandezine-treated MEFs were then fixed for quantification of GFP-LC3 puncta formation. As shown in Figure 5A, 10 μM of hernandezine increased GFP-LC3 puncta formation in Atg7 wild-type MEFs, but not in Atg7-deficient MEFs, indicating the involvement of Atg7 in hernandezine-induced activation of autophagy.

A number of anti-cancer agents have been reported to induce autophagy in various types of cancers [27], however it remains controversial whether autophagy promotes cell death or acts as a pro-survival mechanism. Studies showed that Atg7-deficient MEFs are resistant to induction of autophagy [25]. As hernandezine-induced autophagy required Atg7 (Figure 5A), we therefore utilized both Atg7 wild-type and Atg7-deficient MEFs to determine whether hernandezine-induced autophagy leads to cell death or acts as a pro-survival mechanism [19]. Results showed that hernandezine exhibited less cytotoxicity in Atg7-deficient MEFs when compared to their wild-type counterparts (Figure 5B), similar results were found in HeLa cancer cells with Atg7 knockdown (Figure 5C). These data suggested that hernandezine-induced autophagy could lead to autophagic cell death, because the failure in the induction of autophagy in Atg7-deficient cells suppressed the hernandezine-induced cell death. Hernandezine-induced autophagy requires the involvement of Atg7 and promotes cell death in cancer cells.

### Hernandezine induces autophagic cell death in apoptosis-resistant cancer cells

**Figure 6:**
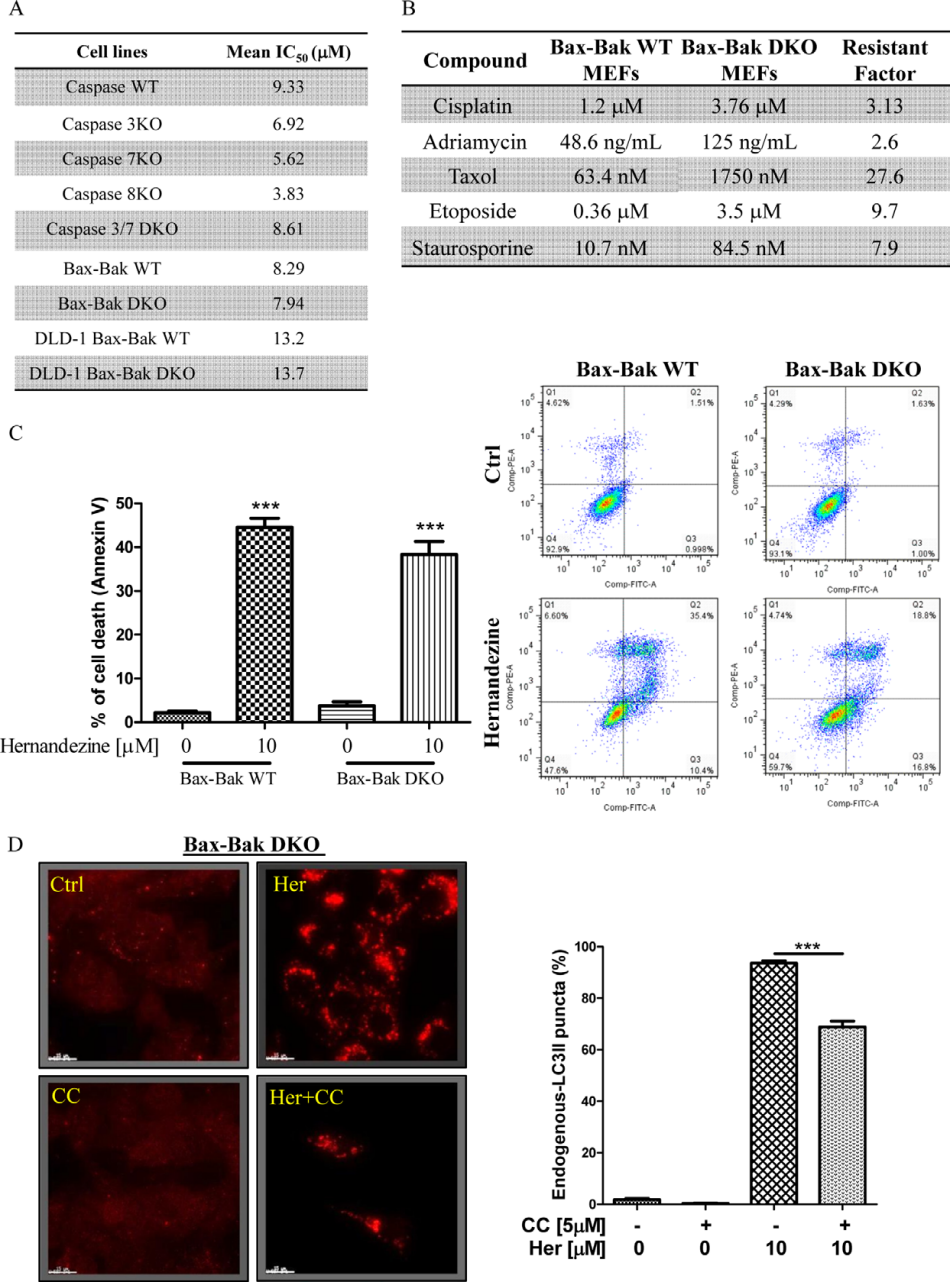
Hernandezine exhibited potent cytotoxicity in apoptosis-resistant cells via autophagy induction (**A**) Cytotoxicity of hernandezine in caspase −3/−7/−8, caspase −3,−7 DKO, Bax-Bak DKO wild-type and deficient MEFs, and DLD-1 Bax-Bak DKO wild-type and deficient colon cancer cells. Both wild-type and deficient cells were incubated with hernandezine at concentrations of 0.19–100 μM for 3 days. Cytotoxicity of hernandezine in wild-type and deficient cells was measured by MTT assay and shown as the mean IC_50_. (**B**) Comparison of multidrug-resistant effects of chemotherapeutic agents in apoptosis-defective Bax-Bak DKO MEFs. Both Bax-Bak WT and Bax-Bak DKO MEFs were treated with cisplatin, adriamycin, taxol, etoposide and staurosporine for 72 h. MTT assay was performed to confirm their cytotoxicity. The IC_50_ values shown on the chart are mean values of three independent experiments. (**C**) Annexin V flow cytometry analysis of hernandezine in Bax-Bak DKO wild-type and deficient MEFs. Both wild-type and deficient MEFs were incubated with DMSO (Ctrl) or 10 μM of hernandezine for 24 h. Hernandezine-induced cell death was then measured by flow analysis after annexin V staining. Data from the bar chart represented the means ± S.D. of cell death (%) from three independent experiments. (**D**) AMPK inhibitor abrogated the hernandezine-induced autophagy in apoptosis-resistant cells. Bax-Bak DKO MEFs were treated with DMSO (Ctrl) or 10 μM of hernandezine with or without 5 μM of the AMPK inhibitor compound C (CC) for 24 h. Hernandezine-induced autophagy were visualised by fluorescence microscopy after staining with the LC3-II antibody followed by TRITC-conjugated anti-mouse secondary antibody. Data from the bar chart represented the means ± S.D. of three independent experiments. ***P < 0.001.

**Figure 7:**
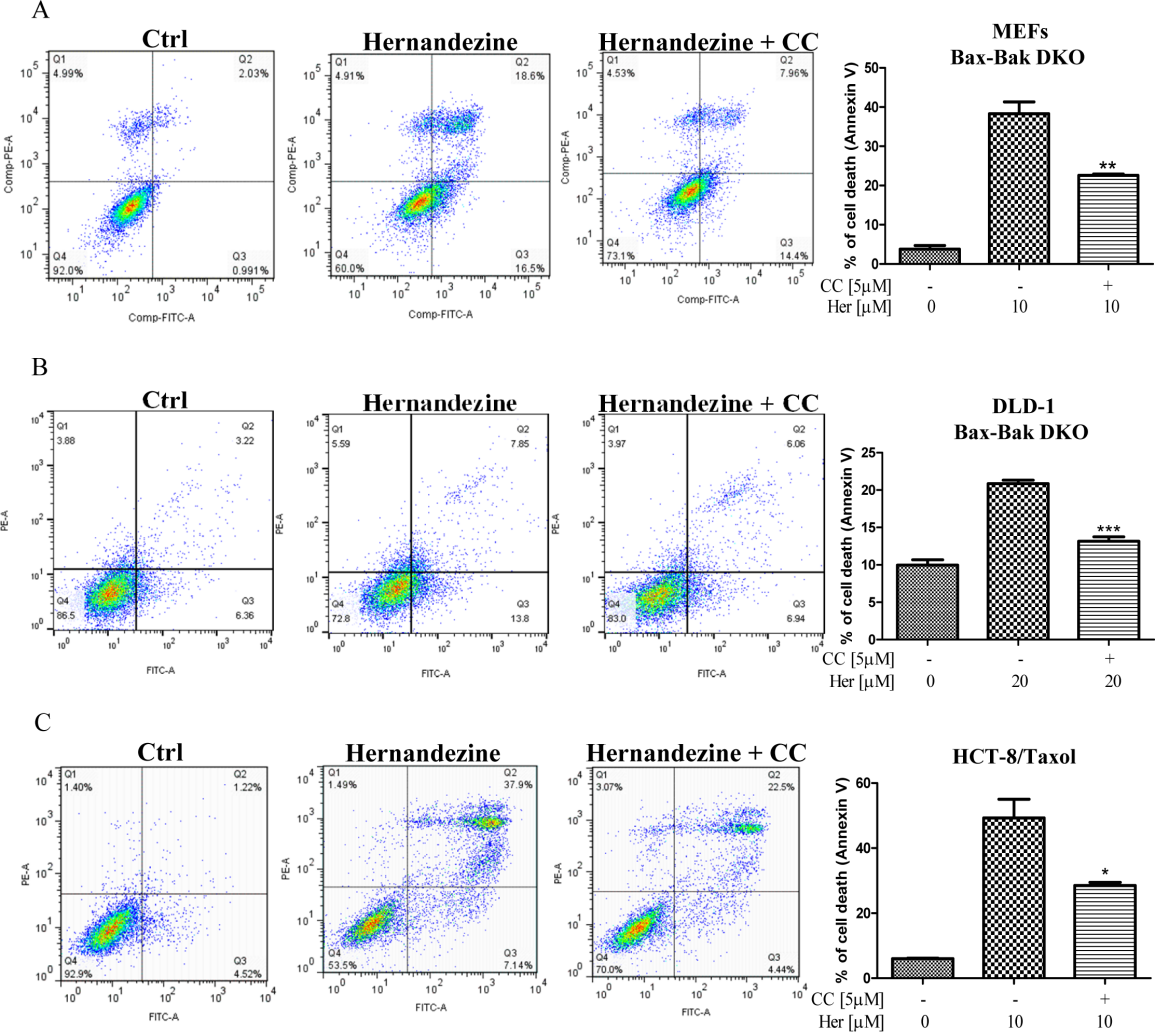
Hernandezine induced autophagic cell death in apoptosis-resistant or drug-resistant cancer cells via AMPK signalling AMPK inhibitor, compound C (CC) abrogated the hernandezine-induced autophagic cell death in apoptosis-resistant and drug-resistant cancers. (**A**) Bax-Bak DKO MEFs were treated with DMSO (Ctrl) or 10 μM of hernandezine with or without 5 μM of the CC for 24 h; (**B**) DLD-1Bax-Bak DKO colon cancer cells were treated with DMSO (Ctrl) or 20 μM of hernandezine with or without 5 μM of the CC for 24 h; (**C**) HCT-8 taxol-resistant colon cancer cells were treated with DMSO (Ctrl) or 10 μM of hernandezine with or without 5 μM of the CC for 24 h. Hernandezine-induced autophagic cell death was measured by flow analysis after annexin V staining. Data from the bar chart represented the means ± S.D. of three independent experiments. *P < 0.05; **P < 0.01; ***P < 0.001. Data from the bar chart represented the means ± S.D. of cell death (%) from three independent experiments.

Cancer cells are frequently resistant to drug-mediated apoptosis [28]. Therefore, the use of small-molecules to induce autophagic cell death in apoptosis-defective or apoptosis-resistant cancer cells may become a promising therapeutic approach [13, 29]. To investigate if the identified AMPK activator hernandezine can exhibit cytotoxic effects towards apoptosis-resistant cells, we adopted a panel of apoptosis-defective or apoptosis-resistant cells such as caspase 3/−7/−8 deficient MEFs, Bax-Bak double knockout (DKO) MEFs and DLD-1 Bax-Bak DKO human colon cancer cells as the cellular models. As shown in Figure 6A, hernandezine demonstrated similar cytotoxic profiles in caspase −3/−7 DKO, wild-type and caspase −3/−7/−8 deficient MEFs. Similar cytotoxic effect towards both Bax-Bak wild-type and DKO MEFs or DLD-1 colon cancer cells were also observed. Bax-Bak DKO MEFs revealed resistance towards chemotherapeutic agents, i.e. cisplatin, adriamycin, taxol, etoposide and staurosporine with resistance factors ranging from 2.6 to 27.6 (Figure 6B), suggesting that hernandezine could circumvent the apoptosis-resistant phenotype of cells conferred by genetic deficiencies. We then examined the cytotoxic effects of hernandezine in both Bax-Bak wild-type and DKO MEFs using annexin V flow cytometry analysis. As expected, there was coherence between the MTT and flow cytometry results, which suggested that hernandezine induces potent cytotoxicity in apoptosis-defective or apoptosis-resistant cells (Figure 6C). Owing to the direct activation of AMPK by hernandezine, we also determined the role of AMPK in hernandezine-induced autophagic cell death in Bax-Bak DKO apoptosis-resistant cells. Consistently, AMPK inhibitor compound C (CC) suppressed the hernandezine-induced autophagy and cell death in Bax-Bak DKO MEFs (Figures 6D and 7A), whereas CC also abrogated hernandezine-induced cell death in DLD-1 Bax-Bak DKO cancer cells (Figure 7B), confirming the key role of AMPK signalling in hernandezine-induced cell death of apoptosis-resistant cancer. Furthermore, the multidrug-resistant (MDR) cancer cells were also adopted to evaluate the potential anti-cancer effect of hernandezine. For this purpose, taxol-resistant HCT-8 colon cancers were incubated with 10 μM of hernandezine in the presence of CC prior to annexin V flow cytometry analysis. Addition of CC blocked the hernandezine-induced cell death in these MDR cancer cells (Figure 7C).

## DISCUSSION

Natural alkaloids namely isoquinoline alkaloids comprising the common structure of isoquinoline nucleus, have been shown to possess anticancer properties as demonstrated by their cytotoxic effect on various types of cancer cells [13]. These alkaloids such as liensinine, isoliensinine, dauricine and cepharanthine, trigger cell death in a non-apoptotic manner, therefore, immortalized cell lines with hampered apoptosis are sensitized to their stimulation. This alkaloid-induced cellular toxicity is associated with the up-regulation of Atg7-dependent autophagy, which is potentially beneficial to anti-cancer therapy [13]. Although the molecular mechanisms underpinning the alkaloid-induced cell death is still elusive, we and others have demonstrated that the AMPK-mTOR signaling cascade is activated by these alkaloids [13, 30–32]. Although other natural compounds such as co-enzyme Q (CoQ), and polyphenol including flavonoids, lignans, stilbenes and phenolic acids were found intertwining with the AMPK signaling pathway [33–35], there is lack of evidence pointing towards the direct activation of AMPK by these compounds. CoQ and polyphenol activate AMPK signaling via the upstream kinases, Ca^2+^–stimulated kinase (CaMKK) and liver kinase B1 (LKB1), which are correlated to their therapeutic potency upon different disease conditions such as obesity, hyperglycemia and insulin resistance [33–35]. Other studies showed that genistein (flavonoid) contributes to obesity control by regulating the transcriptional expression of fatty acid ω-hydroxylase (CYP4F2) through the manipulation of CaMKK [36]. Sauchinone (lignan) activates AMPK phosphorylation by LKB1 kinase, which perturbed the iron-induced oxidative mitochondrial stress and lead to the alleviation of chronic disorders progression [37]. Also, application of CoQ_10_ to the 3T3-L1 adipocytes culture have demonstrated the anti-adipogenic effect of CoQ is mechanistically relevant to the CaMKKβ-AMPK-axis-driven peroxisome proliferator-activated receptor alpha (PPAR-*α*) expression [38].

AMPK activators are ideal pharmacological compounds for cancer therapy, since mTOR kinase that frequently activated in a wide spectrum of tumors are negatively regulated by the LKB1-AMPK pathway [39–41]. Both *in vitro* and animal studies have highlighted the anti-cancer effect of direct activation of AMPK. MT 63–78 (Debio 0903), a direct AMPK activator, thwarts the growth of androgen sensitive and castration resistant prostate cancer cell model (CRPC), and reduces tumor volume of mice intraperitoneally (i.p.) or orally treated with the compound [42]. Recent studies showed that BL-AD008 is a novel dual-target activator of AMPK/ZIPK and induces apoptosis in cervical cancer [43]. Commonly prescribed anti-diabetic drug, metformin, functioning through direct AMPK activation has epidemiologically been proven to downscale the occurrence of pancreas, colon and hepatocellular carcinoma in type 2 diabetic patients [44–47]. Other studies demonstrated that metformin potentiates anticancer effect of dasatinib in head and neck squamous cell carcinoma cells via AMPK-dependent ER stress [48]. It is noteworthy that, metformin has already been approved in clinical trials for the treatment of pancreatic and breast cancers (http://clinicaltrials.gov, IDs NCT01210911, NCT01266486). The association of high glucose uptake with elevated cancer cell proliferation also reinforced the notion of applying AMPK activators clinically for cancer therapy and prevention [49]. Higher mortality rate and cancer risk are linked to the pathological condition of excessive circulatory glucose concentration such as hyperglycemia [50]. AMPK activation in hernandezine-treated cancer cells was induced when the cellular energy state is suppressed, suggesting the close relationship between metabolic glucose anomalies and the pharmacological action of hernandezine towards cancer cell death. However, activation of AMPK may protect cancer cells in response to the micro-environmental stresses that the cancer cells are encountering [51, 52]. Therefore, clinical trials designated to define the optimal clinical stages for AMPK activator application is needed for maximizing their efficacy.

In line with our previous findings that isoquinoline alkaloid is able to induce autophagic cell death in cancer cells [13], hernandezine-induced cytotoxicity is autophagy-dependent. Similar to other isoquinoline alkaloids, hernandezine-induced cytotoxicity is independent of apoptosis. By using a wide spectrum of caspase (−3/−7/−8) and Bax/Bak-deficient cell lines, we concluded that hernandezine may activate autophagic cell death without Bax-Bak or caspases. Apoptotic-related mitochondrial/cytochrome c pathway is frequently disrupted in human cancers and many malignancies [27, 53]. Most chemotherapy-resistant cancers are having defective apoptotic pathways. For example, the Bax-Bak double-knockout MEFs are resistant to various apoptosis-inducing agents [53]. Caspase-3, -8 and -9 are associated with apoptosis-resistance and drug-resistance phenotypes, as well as apoptosis induced by anti-cancer agents [54]. Although caspase-3 activation is crucial to apoptosis, study showed that the induction of apoptosis could be happened in the absence of caspase-3 [55]. Recent studies further highlighted that induction of autophagy for treatment of cisplatin-resistant and p53 mutated cancers [56]. Therefore, novel pharmaceutical interventions inducing cancer cells cytotoxicity through non-apoptotic signaling is inevitable. Hernandezine or generally isoquinoline alkaloids may actually serve more than simply an anti-cancer agent. AMPK is engaged with glucose and lipid metabolisms extensively in different organs and tissues, controlling pancreatic insulin secretion, fatty acid and cholesterol synthesis in liver, lipolysis in adipose tissue, cardiac and skeletal fatty acid oxidation, and glucose uptake [57–60]. Apart from glucose concentration, factors like hormones and cytokines are stimuli of AMPK, illustrating the involvement of multiple pathways in AMPK regulation [61–63]. Therefore, AMPK is positioned at the center of AMPK-mTOR cascade making the kinase the key molecular target for pharmaceutical interventions of different metabolic disorders.

Literatures concerning the direct action of natural compounds on AMPK are scarce. Up to 2012, around 26 patent applications claiming the discovery of direct AMPK activators have been filed [64]. These small-molecules AMPK activators belong to the derivatives of thienopyridone, cyclic benzimidazole, pyrimidine, alkene oxindole and ring-fused imidazole [64]. Accurate examination disclosing that they are reminiscent to each other due to the 4-(2-hydroxypheny)phenyl-side chain and a negatively ionizable group. These structural resemblances seem not to be a mandatory criteria for AMPK activation, because structural modifications of this particular side chain do not induce notable differences in AMPK activation [64]. However, the robust AMPK activation induced by hernandezine, liensinine, isoliensinine, dauricine and cepharanthine agreed with this observation as they do not contain the 4-(2-hydroxypheny) phenyl-side chain. Accordingly, we have proposed a new class of compound exhibiting direct activation of AMPK kinase, and widen the chemical scope for searching new direct AMPK activators.

Isoquinoline alkaloid is a direct activator of AMPK and autophagy, and exhibits its anti-cancer property by inducing cancer cell death. Provided that our candidates are structurally different from other proprietary direct AMPK activators, the present study unveils a novel class of natural small-molecule directly activating AMPK which induces autophagy particularly on apoptotic-resistance cancer.

## MATERIALS AND METHODS

### Chemicals, plasmids and antibodies

All reagents and chemicals were purchased from Sigma (MO, USA) unless otherwise stated. Hernandezine was purchased from China Chengdu Biotechnology Company Ltd. (Chengdu, China) (> 98% purity, HPLC). E64D, pepstatin A, bafilomycin A and compound C were obtained from Calbiochem (Darmstadt, Germany). The pEGFP-LC3 and mRFP–GFP tandem fluorescent-tagged LC3 (tfLC3) plasmids were gifts from Prof. Tamotsu Yoshimori (Osaka University, Japan). Antibodies against LC3B, p-AMPK (Thr172), AMPK, p-p70S6K (Thr389), p70S6K and p-Acc were purchased from Cell Signalling Technologies Inc. (Beverly, MA). The ZyMax^™^ TRITC-conjugated anti-mouse secondary antibodies were purchased from Invitrogen (Scotland, UK). Actin antibody was purchased from Santa Cruz Biotechnology (Santa Cruz, CA).

### Cell culture

All cells were obtained from the American Type Culture Collection (Rockville, MD) unless otherwise specified. Immortalised wild type and Atg7-deficient mouse embryonic fibroblasts (MEF) were kindly provided by Prof. Masaaki Komatsu (Juntendo University, School of Medicine, Japan). Immortalised wild-type and Caspase 3/7-deficient MEFs were gifts from Prof. Richard A. Flavell (Yale University School of Medicine, United States). Immortalised wild type and Caspase 8-deficient MEFs were kindly provided by Prof. Kazuhiro Sakamaki (Kyoto University, Graduate School of Biostudies, Japan). Immortalised wild-type and Bax-Bak double knockout MEFs were kindly provided by Prof. Shigeomi Shimizu (Tokyo Medical and Dental University, Medical Research Institute, Japan). Human DLD-1 Bax-Bak wild-type and deficient isogenic colon cancer cells were purchased from Sigma (MO, USA). HCT-8 taxol-resistant cancer cells were purchased from KeyGEN BioTECH (Shanghai, China). All cells were cultured with medium supplemented with 10% foetal bovine serum (FBS), 50 U/ml penicillin, and 50 mg/ml streptomycin (Invitrogen, Paisley, Scotland, UK). All cell cultures were incubated in a humidified incubator at 37°C with 5% CO_2_.

### Quantification of GFP-LC3 puncta formation

GFP-LC3 puncta were quantified as described previously [26]. Localisation of GFP-LC3 and the fluorescent images were acquired using high magnification widefield epifluorescence microscopy. Images were captured by a Photometrics CoolSNAP HQ2 CCD camera on the Olympus IX71-Applied Precision DeltaVision restoration microscope (Applied Precision, Inc, USA), and the epifluorescence images were numerically deconvolved using DeltaVision algorithms (Applied Precision, Inc.). To quantify autophagy, the percentage of autophagic cells was calculated by counting the number of cells showing increased punctate pattern of GFP-LC3 and dividing by the total number of GFP-positive cells. A minimum of 1000 cells from randomly selected fields was scored per condition per experiment.

### Endogenous autophagy detection

Hernandezine-treated cancer cells on cover slips were fixed with 4% paraformaldehyde (Sigma) and then immersed in methanol for 2 min. Cells were then incubated with anti-LC3 antibody (1:200) in TBST (100 mM Tris HCl, pH 7.5, 150 mM NaCl, 0.05% Tween 20 and 5% BSA) overnight at 4°C. Cells were incubated with anti-mouse secondary antibody (TRITC) (1:200) in TBST containing 5% BSA at 37°C for 1 h in the dark. The coverslips were mounted with FluorSave^™^ mounting media (Calbiochem, San Diego, CA, USA) for fluorescence imaging. Localization of LC3 autophagosomes were captured under the API Delta Vision Live-cell Imaging System (Applied Precision Inc., GE Healthcare Company, Washington, USA). Standard guidelines were followed to monitor autophagy [20]. The percentage of autophagic cells was calculated by counting the number of the cells showing increased punctuate pattern of LC3 fluorescence (≥ 10 dots/cell) in immunofluorescence positive cells over the total number of cells in the same field. A minimum of 1000 cells from randomly selected fields were scored.

### mRFP-GFP tandem fluorescent-tagged LC3 (tfLC3) immunocytochemistry and fluorescence microscopy

HeLa cells were transfected with mRFP-GFP-LC3 for 24 h. After transfection, the cells were treated with hernandezine at 10 μM for 0–24 h. Each correlation plot was derived from the field shown in the fluorescence microscopic image. Colocalization of mRFP with GFP in tfLC3 puncta was measured using ImageJ software, and presented as the percentage of the total number of yellow mRFP+–GFP+ puncta [21].

### MTT cytotoxicity assays

Hernandezine was dissolved in DMSO to a final concentration of 100 mM. Cell viability was measured using the MTT (3-[4, 5-dimethylthiazol-2-yl]-2, 5 diphenyl tetrazolium bromide) assay as described previously [65]. The percentage of viable cells was calculated using the following formula: Cell viability (%) = Cells number _treated_ / Cells number _DMSO control_ × 100. Data were obtained from three independent experiments.

### Flow cytometry analysis

Cell viability and cell death were measured using an annexin V staining kit (BD Biosciences, San Jose, CA, USA). Briefly, cells were treated with 10 μM of hernandezine for 24 h. Cells were then analysed by multiparametric flow cytometry using FITC-Annexin V and Propidium iodide staining (BD Biosciences, San Jose, CA, USA). Flow cytometry was then carried out using a FACSCalibur flow cytometer (BD Biosciences, San Jose, CA, USA). Data acquisition and analysis was performed with CellQuest (BD Biosciences, San Jose, CA, USA). Data were obtained from three independent experiments.

### Western blot analysis

Cells were treated with 10 μM of hernandezine for 24 h at 37°C. After SDS/PAGE electrophoresis, the proteins from SDS/PAGE were electro-transferred to a membrane. The membrane was then immunoblotted with the appropriate antibodies. followed by HRP-conjugated secondary antibody for 60 min. Band intensities were quantified with ImageJ (N.I.H.).

### AMPK kinase assay

AMPK kinase assay was performed using CycLex^®^ AMPK Kinase Assay Kit (MBL, Japan) according to manufacturing instructions. In brief, 0.2 ng of AMPK (α1/β1γ1) active enzyme (CycLex Co., Ltd.) was incubated in well with 10X of hernandezine (50 & 100 μM) or 10X of positive control, AMP (100 μM) in kinase assay buffer (50 μM ATP & 10 mM DTT) at 30°C for 20 min. The reaction was then stopped by washing with buffer for 5 times. Then, anti-phospho-mouse IRS-1 S789 monoclonal antibody was added to each well at room temperature for 30 min. After washing with buffer for 5 times, HRP-conjugated anti-mouse IgG was added to each well at room temperature for 30 min. After washing with wash buffer, the TMB substrate reagent was incubated in wells at room temperature for 5–15 min. Stop solution was added to each well before measuring absorbance at 450/550 nm.

### Statistical analysis

The results were expressed as the means ± SD as indicated. Differences were considered statistically significant when the *P*-value was less than 0.05. Student's *t*-test or one-way ANOVA analysis was used for comparison among different groups.
